# Supplementary material for: People’s naïve belief about curiosity and interest: A qualitative study
Source: PLoS One. 2021 Sep 30;16(9):e0256632. doi: 10.1371/journal.pone.0256632 (PMC8483368; doi:10.1371/journal.pone.0256632)
Supplement: S1 Appendix — (DOCX) [file pone.0256632.s001.docx]

**Appendixes**

**Coding process**

Braun and Clarke (2006) argued that there are six phases for thematic analysis: (1) Data familiarization; (2) Producing initial codes; (3) Seeking themes; (4) Theme review; (5) Finalizing theme names and definitions; and (6) Reporting. We followed these steps to do thematic analysis. Specifically, in the first phase (i.e. data familiarization), **t**he computer program NVIVO was selected to conduct the thematic analysis electronically. Then, reading and re-reading took place to become familiar with the data. After that, initial opinions and thoughts were noted in accordance with research questions. In the second phase (i.e., producing initial codes), a systemised approach to coding was used, in accordance with the principals of the constant comparison method in which data are coded and re-coded iteratively and inductively. As for the third phase, the initial codes were revised and regrouped to create more definitive groups, and common categories were finalised and unified around central themes. In the fourth phase, the themes were checked to see if they were coherent and meaningful, and worked at both Level 1 (the coded extracts) and Level 2 (the entire data set). After reviewing the themes, names were generated for each theme to ensure they captured the meaning and clearly contributed to a consistent overarching interpretation of the data in the fifth phase. In the last phase, clear and vivid quotes were selected that best represented or illustrated a particular theme.

See Results section for the final themes and the quotes. As an example for the data analysis process, you can see an interim thematic map before the final themes below.
