# Supplementary figures and images for: People’s naïve belief about curiosity and interest: A qualitative study
Source: PLoS One. 2021 Sep 30;16(9):e0256632. doi: 10.1371/journal.pone.0256632 (PMC8483368; doi:10.1371/journal.pone.0256632)

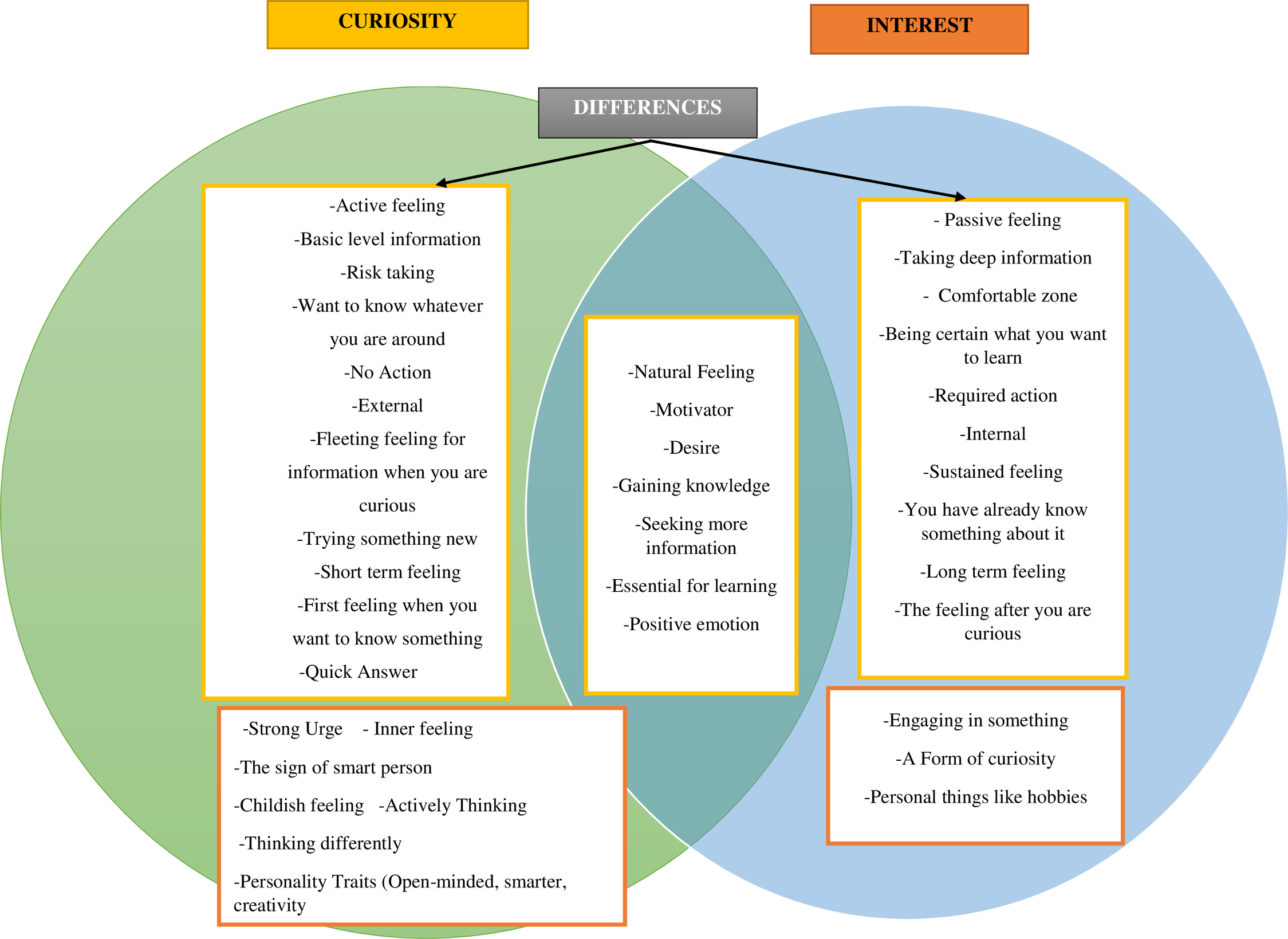

Supplement: S2 Appendix — (TIF) [file pone.0256632.s002.tif]
