# Supplementary material for: People’s naïve belief about curiosity and interest: A qualitative study
Source: PLoS One. 2021 Sep 30;16(9):e0256632. doi: 10.1371/journal.pone.0256632 (PMC8483368; doi:10.1371/journal.pone.0256632)
Supplement: Attachment — Submitted filename: R2RPONE-D-20-25903.docx [file pone.0256632.s003.docx]

************************************************************************Responses to Editor**

**************************************************************************

*PONE-D-20-25903*

*People’s naïve belief about curiosity and interest: a qualitative study*

*PLOS ONE*

*Dear Dr. Aslan,*

*Thank you for submitting your manuscript to PLOS ONE. After careful consideration, we feel that it has merit but does not fully meet PLOS ONE’s publication criteria as it currently stands. Therefore, we invite you to submit a revised version of the manuscript that carefully and systematically addresses all the points raised by the two reviewers of your manuscript during the review process. Both reviewers provide detailed points that you need to address satisfactorily. Please pay particular attention to establishing a more convincing rationale for this research (especially in relation to pertinent previous research), and ensuring that the research aims that you indicate and the methods you use correspond to each other (Reviewer 2 has noted a number of serious problems with the method you used). You also need to ensure that explanations and descriptions of the qualitative analysis procedures you used are sufficiently understandable even to those who do not possess expertise in those procedures.*

*Please submit your revised manuscript by Jan 11 2021 11:59PM. If you will need more time than this to complete your revisions, please reply to this message or contact the journal office at plosone@plos.org. When you're ready to submit your revision, log on to https://www.editorialmanager.com/pone/ and select the 'Submissions Needing Revision' folder to locate your manuscript file.*

*Please include the following items when submitting your revised manuscript:*

*A rebuttal letter that responds to each point raised by the academic editor and reviewer(s). You should upload this letter as a separate file labelled 'Response to Reviewers'.*

*A marked-up copy of your manuscript that highlights changes made to the original version. You should upload this as a separate file labelled 'Revised Manuscript with Track Changes'.*

*An unmarked version of your revised paper without tracked changes. You should upload this as a separate file labelled 'Manuscript'.*

*If you would like to make changes to your financial disclosure, please include your updated statement in your cover letter. Guidelines for resubmitting your figure files are available below the reviewer comments at the end of this letter.*

*If applicable, we recommend that you deposit your laboratory protocols in protocols.io to enhance the reproducibility of your results. Protocols.io assigns your protocol its own identifier (DOI) so that it can be cited independently in the future. For instructions see: http://journals.plos.org/plosone/s/submission-guidelines#loc-laboratory-protocols*

*We look forward to receiving your revised manuscript.*

*Kind regards,*

*Emmanuel Manalo, PhD*

*Academic Editor*

*PLOS ONE*

**Response:** We would like to thank you for your helpful comments. We have addressed all the comments from the reviewers, including the major points you raised. Please see our responses to specific comments from reviewers.

Regarding the protocol for reproducibility, given the qualitative nature of the study, it is difficult to create such a protocol. In fact, qualitative studies are typically conducted to generate and inspire hypotheses (which we did), rather than to confirm specific hypotheses (which clearly needs a reproducible protocol). Therefore, we are afraid that we cannot deposit our protocol.

************************************************************************

**Reviewer #1**: *I enjoyed reading about this study and found it to present interesting data that researchers could use in considering the constructs of curiosity and creativity in future work. One challenge in thinking about the paper’s usefulness, however, was the framing of the work and the discussion of specific ways that it could inform future work. Specifically – why is it useful to know what laypeople think about curiosity and interest, and in what ways can researchers use the themes identified to inform their work? Or – perhaps the paper could be reframed to more directly present the goals of simply understanding how people view curiosity and interest? This latter suggestion seems like one that makes sense to me but is not aligned with the current framing of the literature reviewed and discussion of the results. There are many papers on teachers’ perceptions of student characteristics that do this, and I can think of papers that look at perceptions of curiosity, which is not the same as what this study did but similarly looks at the “layperson perspective”. A couple that come to mind are Kashdan’s paper on how curious people are viewed, and Post’s paper on conceptions of curiosity (citations provided below).

Overall, I think this paper presents interesting data but would be strengthened by reframing the intro around clearer goals of what is learned and how this new knowledge can be used, and then continuing to describe how the knowledge advances specific literature and can be used in different ways in the discussion. I describe a specific suggestion and/or questions related to each section below.*

**Response:** Thank you very much for your comments and the references. We have substantially changed the latter part of the introduction to clarify the rationale of why we should examine lay people’s definitions of curiosity and interest (page 5-6). We have also incorporated the citations that the reviewer suggested (page 6, line 202-203).

**********************************************************************

**Reviewer 1:**

*Introduction:*
*After reviewing prior definitions and theories of curiosity and interest in some depth, with an emphasis on the lack of agreement in definitions and measures, the researchers discuss the reward-learning framework and the purpose of the study: to understand the culturally agreed-upon commonalities and differences between curiosity and interest in layperson definitions. While I believe this purpose is interesting in terms of comparisons to and understanding how scientific research has defined and measured curiosity and interest, it seemed strange that the introduction did very little in terms of discussing prior work that has looked at layperson definitions of these constructs, and why it matters how people outside of the scientific research community define them. The challenge of advancing research without common definitions is given, but what is the value in understanding the layperson definitions? I can imagine many possible answers to this and expect that there is prior research that could be discussed to explain, beyond what is given as justification for the methods used. What is the value of studying naive beliefs?*

**Response:** We have revised our introduction to clarify the reason why we examined lay people’s definitions of curiosity and interest, according to the reward-learning framework of knowledge acquisition (page 5-6, line 158-182). To our knowledge, except for a few studies that the reviewer suggested (which we really appreciate), there is little work that has directly examined lay people’s perceptions of curiosity and interest. We have clarified this point on page 6, line 183-197.

**********************************************************************

**Reviewer 1:**

*Methods:*
*The authors state that they planned to collect 150 participants before examining the data but seemed to have stopped at 135 at which point the data already showed thematic saturation – this was unclear (why were the data examined early?).*

*I am not a qualitative researcher and appreciated the information about the qualitative methods for identifying themes.*

**Response:** Thanks for your comment. We are sorry for the lack of clarity. We first recruited 135 participants and intended to collect more data if thematic saturation did not occur. We did not examine the data early. We have clarified this point on page 8, line 271-273.

**********************************************************************

**Reviewer 1:**

*Results:*
*The diagram was helpful in understanding the structure and organization of responses within the identified themes, and the quoted examples were helpful in demonstrating the themes. I did wonder how common different types of responses were and how variable the responses were. For example, what portion of responses fell into each of the different themes, or maybe it makes more sense to know what portion of responses were outside of those themes? I imagine a single person’s response could touch on multiple themes, but I wondered how many people had responses that were outside of the identified themes, and perhaps how many touched on only one whereas how many were more inclusive of all of the different subthemes? Were there any patterns related to individual differences such as gender, age, or other background characteristics?*

**Response:** The spirit of qualitative study is not to count the number of relevant responses. The use of numbers can lead to thinking of these themes in terms of variance, in a quantitative framework. This undercuts the main advantages of qualitative research and can lead to linear assumptions being drawn on consequences, causes and relationships in the data. This is not the purpose of qualitative exploration like this and therefore we have not included reports on proportion and numbers. Even if there are only a few relevant responses, if they are inspiring enough to generate a theme, they should be incorporated in describing the results. That being said, we have now included more demographic information within the Participant section (page 7-8, line 256-260) to provide more contextual information about the survey, and the sample as a whole.

**********************************************************************

**Reviewer 1:**

*Discussion:*

*I appreciated the contrasting between layperson definitions of curiosity and interest and the ways these have been defined in scientific research in the discussion, but I still felt like the paper could be strengthened with a clearer framing of what can be done with the knowledge learned from this work. The authors state, “Participants’ interpretations of the terms in their daily life can decrease practically unnecessary confusion regarding the definition of curiosity and interest in applied science (e.g. education, psychology, and neuroscience). This will help researchers to understand and consider peoples’ naïve belief about curiosity and interest when designing experiments in this field.” In what ways can the data collected and themes that emerged decrease this confusion? What does that look like – what should future research do as a result of knowing this? How will this help with designing future experiments?*

**Response:** Like the Introduction, we have revised the Discussion section to clarify the value of the current work.

**********************************************************************

**Reviewer 2:** *The objective of the present research is to examine lay conceptions / naïve beliefs about curiosity and interest. I really like the idea of that research. That said, I do have questions and serious concerns.*

**Theory**
*1.) l. 49-50: “lack of consensus about how researchers should conceptualize these terms”. I don’t agree with that conclusion. Apart from that I think that it is necessary to underline that with literature.*

**Response:** We would like to thank you for your helpful comments. We have now added references to literature that mentions the same point, i.e., that there is a lack of consensus (page 2, line 48-50)

**********************************************************************

**Reviewer 2:**

**Theory**

*2.) l. 62-64: As a reader I would like to have some literature supporting the statement.*

**Response:** We have omitted this sentence as it may be confusing.

**********************************************************************

**Reviewer 2:**

**Theory**

*3.) l. 85-86: “curiosity is conceptualized as immediate experience in response to stimuli in the external environment”: I don’t agree with that conclusion. At least epistemic curiosity is elicited also by conceptual problems, puzzling ideas and so on. This has clearly an internal focus. Also, Berlyne distinguished between specific and diverse curiosity behavior. Diverse curiosity behavior is an explorative behavior that is executed because of absent stimulation.*

**Response:** We agree that some researchers would disagree (which is the point of our work). We have clarified that this is just the perspective of some researchers, not all researchers (page 3, line 99).

**********************************************************************

**Reviewer 2:**

**Theory**

*4.) I have serious concerns about your theoretical reasoning. To my opinion, your reasoning for the research question is not sufficiently elaborated: Why would you expect differences in the lay conceptions between curiosity and interest? May be people just use it interchangeably? What exactly is the additional value in knowing the naïve beliefs of lay people? Lay conceptions also have their pitfalls (not explicit, consistent, parsimonious and productive enough).*

**Response:** We have substantially revised the latter part of the introduction to address this issue, basing our approach on a theoretical framework (reward-learning framework of knowledge acquisition). We believe that our decision to examine lay beliefs is now sufficiently justified. We have also discussed the pitfalls of relying on self-report on page 4-5-6, line 129-197.

**********************************************************************

**Reviewer 2:**

**Theory**

*5.) l. 136: Why there is an inherent impossibility to define these terms in a scientific manner?*

**Response:** We have revised the manuscript to clarify this point (page 5-6, line 158-197)

**********************************************************************

**Reviewer 2:**

**Method**
*6.) There is discussion about the quality of data gained through MTurk (Chmielewski & Kucker, 2019; Buhrmester, Talaifar & Gosling, 2018). I think you should at least discuss possible impact on data quality; also consider how that discussion applies to qualitative data.*

**Response:** Thanks for your comment. We now state that we implemented data screening to ensure the data quality obtained via MTurk (Method section, on page 7, line 239-252). In addition, we have added a discussion on how this issue applies in the context of qualitative research in the General Discussion (page 25-26, line 725-768).

**********************************************************************

**Reviewer 2:**

**Method**

*7.) I wonder whether you have also captured educational background and English literacy. Answers on open question certainly depend on the eloquence of participants.*

**Response:** We have revised our manuscript incorporating this point in the Participants section. (page 8, line 256-260)

**********************************************************************

**Reviewer 2:**

**Method**

*8.) l. 169: you mentioned pilot data? What kind of pilot study did you conduct?*

**Response:** We are sorry for the lack of clarity. ‘Pilot study’ only referred to the initial sampling of a few participants to ensure that the study was viable. The procedure was the identical. We have now clarified this point on page 7, line 239-251.

**********************************************************************

**Reviewer 2:**

**Method**

*9.) I have serious concern about the fit between your research aim and the method you apply, or more specifically the question you ask the participants:*
*- In line 135 – 136 you mention that “people have intuitive, but somehow agreed-upon understanding of how they are different”. However, if you target the intuitive concepts of people it is not appropriate to as them for an explicit definition. In my opinion you should than analyze how people are using these two concepts in their everyday language.*
*- People don’t have a ready definition of interest and curiosity in their mind.*
*- In the discussion you emphasize that “the use of open-ended questions ensured that no conceptual priming took place” (l. 483). I strongly disagree with that. By posing one question for curiosity and one for interest you imply that these are two different things. And you intensify that by asking about similarities and differences.*

**Response:** We completely understand your concerns. Thank you very much for these great points. Regarding the first and the second points (they are related questions), we agree, and we now discuss this limitation in General Discussion. We are indeed trying to address the issue using an ecological momentary assessment as a complementary approach, and we have cited this work as an example. With regards to the last point, we never told participants to produce different definitions. In addition, participants could say that they were identical when being asked the similarities and differences (we have clarified this point in the method section). However, none of the participants indicated that curiosity and interest are identical. Some participants said that the terms have the same meaning in terms of emotion and seeking information, but did not say that they were identical in every aspect. In addition, in an independent sample we collected, using a 5 point Likert scale asking the similarity and differences of curiosity, only 1% of participants said they are completely similar. We have added these points to the methods section. Also, we have omitted the sentence that the reviewer was concerned about.

**********************************************************************

**Reviewer 2:**

**Method**

*10.) As a reader I like to know more about the answers given: How much did the participants write? did everybody answered to every question? Did somebody write that the concepts are the same?*

**Response:** Participants wrote a minimum of 80 words for each question. Their responses were between 85 and 150 words. Six participants answered only one part of the question (e.g., the meaning of interest or curiosity only). There were no participants who indicated that the concepts are the same. We have incorporated all this information on page 9, line 298-306.

**********************************************************************

**Reviewer 2:**

**Method**

*11.) I think more information about the coding process are necessary. How did you come to your themes? As a reader I would like you to illustrate the different steps with examples. Until now the reproducibility is low.*

**Response:** We have tried to clarify the coding process in the main text, and added a table (see Appendix) that details the steps taken to derive themes (page 10-12, line 313-364). We have also checked other qualitative studies (e.g., Altun, 2019; McLean & Griffiths, 2018; Watson, et al., 2019; Beng et al., 2015) and our description of the coding process is quite comparable with (or more detailed than) these studies.

**********************************************************************

**Reviewer 2:**

**Method**

*12.) Are there any parameters that provide information about the quality of the coding process?*

**Response:** We took two measures to ensure the quality of coding. Firstly, during the third phase of thematic analysis from Braun and Clarke (2006), the coder (first author) had a meeting with other researchers (fourth and fifth authors) who were not involved with the coding, in order to discuss the appropriateness of the coding and themes. Second, the fourth phase of thematic analysis from Braun and Clarke (2006) was conducted to review mind maps and themes in light of the data. Please see the Appendix for more information about the steps we have taken to conduct the thematic analysis.

**********************************************************************

**Reviewer 2:**

**Method**

*13.) l. 205 what does “line by line coding” mean? Is it a sentence?*

**Response:** Yes. This is the same as the term “sentence by sentence” but in the context of qualitative analysis it is commonly called “line by line” coding. We have clarified this point on page 10, line 316-349.

**********************************************************************

**Reviewer 2:**

**Method**

*14.) What do you mean by “we coded the explicit and implicit meaning for all data”? please give an example.*

**Response:** We have now clarified this using an example (page 10, line 316-349).

**********************************************************************

**Reviewer 2:**

**Results**

*In general, I have difficulties understanding the results. The meaning or content of the themes and subthemes are not sufficiently clear and precisely described.*
*15.) If I understood right: in the first theme curiosity and interest is ordered along two dimensions, namely, passive – active and stable – fleeting. Is that correct?*

**Response:** We did not intend to present a two-dimensional structure. We agree that stable and active are not necessarily two ends of the same continuum and so we do not need to put curiosity and interest at the opposite ends of a continuum. From the data, we simply derived that curiosity is perceived as active whereas interest feels stable. There was no indication of dimensionality.

**********************************************************************

**Reviewer 2:**

**Results**

*6.) In l. 280 – 283 you equate passive with stable. I don’t see in your quotation why that should be like that? (l. 231-232)*

**Response:** This point also relates to the coding process. (pages 10-11, line 329-349 in the Data Analysis). We now include four quotes with our interpretations to clarify this point (page 12-13, line 384-394 and page 15-16, line 454-467).

**********************************************************************

**Reviewer 2:**

**Results**

*17.) I sometimes don’t understand how the quotations fit to theme or sub-theme (e.g. sub theme l. 311 -320: these quotations don’t fit to the part positive emotion?)*

**Response:** We have added one more quote in the example provided by the reviewer (page 17, line 498-506). We believe that, in general, participants’ quoted responses touch upon positive emotions and natural feelings from their daily life.

**********************************************************************

**Reviewer 2:**

**Results**

*18.) L. 378: “curiosity is more related to the pursuit of basic and immediate knowledge”; what do you mean by basic? In the sense of fundamental? Or in the sense of simple?*

**Response:** Yes, we are sorry. We meant simple answers or knowledge. We have now clarified this (page 20, line 566).

**********************************************************************

**Reviewer 2:**

**Results**

*19.) L. 393 – 395: I don’t understand that paragraph.*

**Response:** We apologize. We have now clarified the paragraph (page 21, line 584-588)

**********************************************************************

**Reviewer 2:**

**Results**

*20.) Your figure is difficult to read (it is pixelated).*

**Response:** Thank you for bringing this to our attention, we have now replaced the figure with a higher-quality version.

**********************************************************************

**Reviewer 2:**

**Discussion**
*I find it a bit difficult to evaluate the discussion, as I am not really clear with the results.*

*21.) In my opinion the authors should broaden the limitation part and discuss about the*
*- possible data quality constraints due to mTurk*
*- implicit theories, lay theories are known for contrariness, inconsistencies, lack of explicitness and so forth. You should discuss about that in relation to your data (e.g l. 290 “passive action”; what is meant by child-like emotion?)*

**Response:** We have expanded the limitations section for these issues according to the reviewer’s helpful suggestions. These issues are discussed on page 15-16, line 454-467 page 23, line 640-643 and page 25.

**********************************************************************

**Reviewer 2:**

**Discussion**

*22.) How does the knowledge gained through the present study does contribute to the differentiation between interest and curiosity from the scientific perspective?*

**Response:** This point has now been clarified in the Introduction as well as in the General Discussion.

**********************************************************************

**Reviewer 2:**

**Discussion**

*23.) l. 440-442: as far as I know the literature, for adults and for children curiosity does not arise if something is too new, complex etc. and it does not arise when it not new, complex etc. at all.*

**Response:** We have omitted the relevant paragraph in the course of refining the manuscript.

**********************************************************************

**Reviewer 2:**

**Discussion**

*24.) l. 449 – 450: “more intense attentional focus than curiosity”: in the results interest is described as passive; how does that matches?*

**Response:** We have now clarified the part (page 23, line 653-656)

**********************************************************************
