# Supplementary material for: People’s naïve belief about curiosity and interest: A qualitative study
Source: PLoS One. 2021 Sep 30;16(9):e0256632. doi: 10.1371/journal.pone.0256632 (PMC8483368; doi:10.1371/journal.pone.0256632)
Supplement: Attachment — Submitted filename: Response to Reviewers.docx [file pone.0256632.s004.docx]

************************************************************************Responses to Editor**

**************************************************************************

*PONE-D-20-25903R1*

*People’s naïve belief about curiosity and interest: a qualitative study*

*PLOS ONE*

*Dear Dr. Aslan,

Thank you for submitting your manuscript to PLOS ONE. After careful consideration, we feel that it has merit but does not fully meet PLOS ONE’s publication criteria as it currently stands. Therefore, we invite you to submit a revised version of the manuscript that addresses the points raised during the review process. In particular, we would like you to carefully and systematically address ALL the outstanding issues detailed by Reviewer 2 below.

Please submit your revised manuscript by Aug 01 2021 11:59PM. If you will need more time than this to complete your revisions, please reply to this message or contact the journal office at* [*plosone@plos.org*](mailto:plosone@plos.org)*. When you're ready to submit your revision, log on to* [*https://www.editorialmanager.com/pone/*](https://www.editorialmanager.com/pone/) *and select the 'Submissions Needing Revision' folder to locate your manuscript file.

Please include the following items when submitting your revised manuscript:*

- *A rebuttal letter that responds to each point raised by the academic editor and reviewer(s). You should upload this letter as a separate file labeled 'Response to Reviewers'.*
- *A marked-up copy of your manuscript that highlights changes made to the original version. You should upload this as a separate file labeled 'Revised Manuscript with Track Changes'.*
- *An unmarked version of your revised paper without tracked changes. You should upload this as a separate file labeled 'Manuscript'.*

*If you would like to make changes to your financial disclosure, please include your updated statement in your cover letter. Guidelines for resubmitting your figure files are available below the reviewer comments at the end of this letter.

If applicable, we recommend that you deposit your laboratory protocols in* [*protocols.io*](http://protocols.io/) *to enhance the reproducibility of your results. Protocols.io assigns your protocol its own identifier (DOI) so that it can be cited independently in the future. For instructions see:* [*http://journals.plos.org/plosone/s/submission-guidelines#loc-laboratory-protocols*](http://journals.plos.org/plosone/s/submission-guidelines#loc-laboratory-protocols)*. Additionally, PLOS ONE offers an option for publishing peer-reviewed Lab Protocol articles, which describe protocols hosted on* [*protocols.io*](http://protocols.io/)*. Read more information on sharing protocols at* [*https://plos.org/protocols?utm_medium=editorial-email&utm_source=authorletters&utm_campaign=protocols*](https://plos.org/protocols?utm_medium=editorial-email&utm_source=authorletters&utm_campaign=protocols)*.

We look forward to receiving your revised manuscript.

Kind regards,

Emmanuel Manalo, PhD
Academic Editor
PLOS ONE*

**Response:** We would like to thank you for your helpful comments. We have addressed all the comments from the reviewers. Please see our responses to specific comments made by the reviewers below.

************************************************************************

**Reviewer 1**:

*Reviewer #1: Thank you for the responses to my questions and your work revising this manuscript. I think it will be of interest to many others, and I also value having more qualitative research on curiosity.*

**Response:** Thank you for the positive feedback.

**********************************************************************

**Reviewer 2**:

*Reviewer #2: Comments to the authors*

*Thanks for addressing the issues raised. I think the manuscript has become better. That said, I still have questions and concerns.*

*Theory

I still have concerns about your theoretical reasoning.

1.) I think you should explain the reward-learning framework of knowledge acquisition. Otherwise the respective paragraph is hard to understand.*

**Response:** Thank you very much for your comments. We have revised the text and added a paragraph regarding this (page.5).

**********************************************************************

**Reviewers:**

*2.) I don’t understand how the reward-learning framework of knowledge acquisition helps to explain why it is so difficult to define curiosity and/or interest? I mean a lot of our psychological constructs have been “out there” before psychology started to study them; that, however, does not mean that we don’t have agreed upon definition.*

**Response:**

Thanks for your feedback. Our point is not that we do not have agreed-upon definitions. Our point is that we cannot *decide* which definition is the right definition. We can have an agreed-upon definition if everyone agrees, but when there are two (or more) different perspectives on how to define them (as is the case in the current literature), it is not possible to decide which is right because they are subjective. We have clarified this point on page 5. Note that this perspective is not radical or peculiar --- this is one of the major perspectives in cognitive science or philosophy of mind. We have also noted this in the same paragraph.

**********************************************************************

**Reviewers:**

*3.) p. 6: I understand your reasoning that if people construed two different terms that it seems likely that there are also two different underlying concepts (even though one could surely also argue that the more important a concept is the more words are construed for that specific concept). Anyway, I would not go with you that they are qualitatively different and are supported by qualitatively different psychological processes. You could call a person bright, brainy, clever, and/or intelligent; and these words certainly have slightly different connotation, however, they are not something totally different, and are probably not supported by qualitatively different psychological processes*.

**Response:** We would like to thank you for your helpful comments. We agree that it was an overstep to say that having two different language suggests they are “qualitatively” different. Yes, it is entirely possible that there is some overlap. We have omitted the word “qualitative” and rephrased the sentences on page 6 and throughout the manuscript.

**********************************************************************

**Reviewers:**

*4.) P. 6: Even though I understand you’re reasoning that the somehow “fresh” view of lay people may bring some new insights in the discussion, I wonder why you expect that the scientific definitions of interest and curiosity substantially deviate from lay perception of these concepts?*

**Response:** We do not strongly expect that this is the case and honestly we do not know. This was an “if” statement (“IF scientific definitions and naïve theories strongly deviate…”). In the revised manuscript, we rephrased the sentence to clarify that this is an if statement, not an expectation (page 7).

**********************************************************************

**Reviewers:**

*5.) We know from psychological research that lay beliefs and implicit theories have real-life consequences (e.g. implicit theories about intelligence, illness perceptions). Could you imagine something in the same line?*

**Response:** This is a great point. We have now noted this on page 7.

**********************************************************************

**Reviewers:**

*6.) p.7: What are the results of the studies examining people’s perception about curiosity/interest? Is there anything to infer for the results of the present study? Are the results somehow similar to the results reported here?*

**Response:** We have added explanations about the studies (page 7-8) and suggestions for the current study. We have noted that Post et al., (2018) is especially informative in discussing our findings. As such, we have added a discussion point in relation to this study in General Discussion. However, there has been little research that directly compared the definitions of curiosity and interest.

Note that we have recently published a paper (while revising the current manuscript) that directly examined people’s naive beliefs about curiosity and interest using a machine-learning method (not qualitative analysis). Some of the data overlaps. We already noted this in the original manuscript but have now revised the citation as the paper has just been published.

**********************************************************************

**Reviewers:**

*Method
7.) I still have concerns about your methods. I would not expect everybody to say that curiosity and interest are the same concepts. However, I wonder if you would have asked different groups of people about curiosity and interest whether the differences would be so pronounced. And I would also expect that you find more commonalities.*

*8.) Did you randomize the first question?*

**Response:** Unfortunately, we did not randomize the first two questions. This point is now clarified on page 11. We agree that this methodological issue raises the concern that the reviewer mentioned. We have explicitly noted this point in General Discussion.

**********************************************************************

**Reviewers:**

*9.) As far as I could see also in the independent data you cite, people were asked for a definition of curiosity and interest. So actually we have the same situation.*

**Response:** Yes, the study examined the same situation. We have clarified this point on page 11. We also understand that this means that this information is not a perfection solution to the concern that the reviewer raised. Again, we have provided discussion on this issue on page 27.

**********************************************************************

**Reviewers:**

*10.) Is there anything like Kappa, interrater reliability?*

**Response:** As indicated in the previous response, qualitative research does not normally (though there are exceptions) concern the “number of counts” as the purpose is to generate hypotheses rather than confirm a theory. Inter-rater reliability or similar metric does not fit with the purpose --- the idiosyncratic interpretation of researchers is what is valued in qualitative research. Therefore, it goes against the philosophy of qualitative methods to use such reliability metric. We have clarified the purpose of qualitative research method at the end of the introduction (page 8).

**********************************************************************

**Reviewers:**

*Discussion
11.) P. 22 You mentioned that the results suggest that the human knowledge-acquisition process may be organized in two different part/stages. What parts of the model/framework do you mean? Or don’t you mean the framework? Could you elaborate more on that.*

**Response:** This is based on our results but this observation is also consistent with the framework. We have clarified this on page 26. In response to an earlier comment, we have also included detailed explanations about the framework (page 5) and accordingly, we believe this point is much clearer now. We feel that this part is a bit speculative, especially given the exploratory nature of the study. As such, we have moved this part toward the end, explicitly noting that this is a speculative thought.

**********************************************************************

**Reviewers:**

*12.) If you do not mean the framework – could you please discuss your results with respect to the model? To what parts of the model do the lay concepts relate?*

**Response:** We have also clarified how the results map onto the framework on page 26.
